# Supplementary figures and images for: A Large and Phylogenetically Diverse Class of Type 1 Opsins Lacking a Canonical Retinal Binding Site
Source: PLoS One. 2016 Jun 21;11(6):e0156543. doi: 10.1371/journal.pone.0156543 (PMC4915679; doi:10.1371/journal.pone.0156543)

A

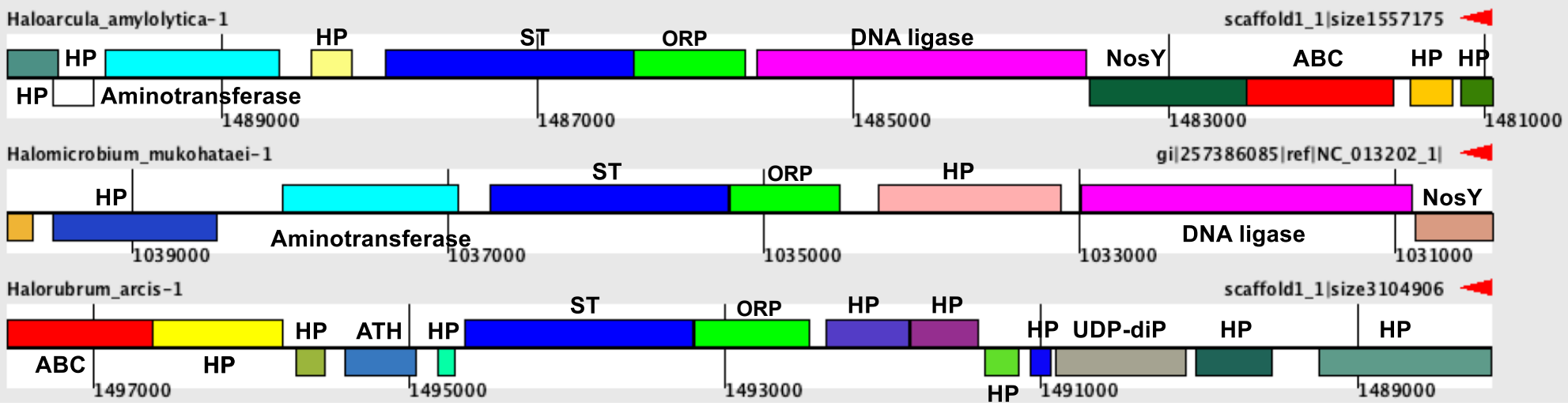

B

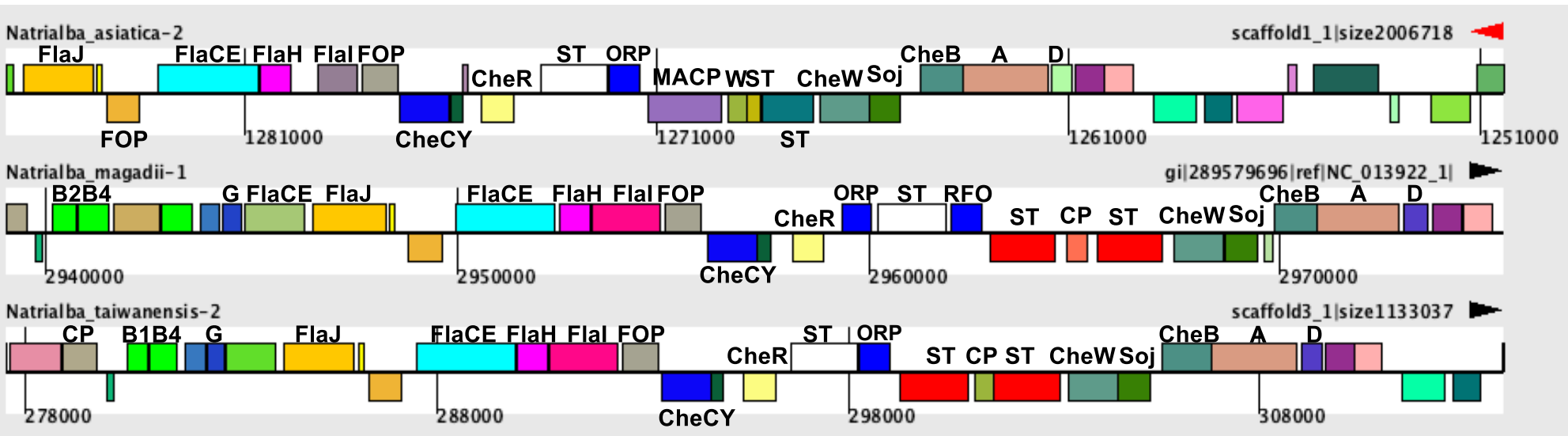

Supplement: S1 Fig — Representative genomic contexts of Group A (A) and Group B (B) ORPs. Genome context was visualized in JContextExplorer [47]. HP = hypothetical protein/unknown, Aminotransferase = serine-pyruvate aminotransferase/archaeal aspartate aminotransferase, ST = signal transducer, ORP = opsin-related protein, ABC = ABC transporter ATP-binding protein, ATH = acyl-CoA thioester hydrolase, UDP-diP = undecaprenyl-diphosphatase. B1/2/4 = Flagellin B1/B2/B4 precursor, FOP = conserved fla operon protein, MACP = methyl-accepting chemotaxis protein, W = CheW, G = FlaG, CP = conserved che operon protein/chemotaxis protein, Soj = sporulation initiation inhibitor protein. (PDF) [file pone.0156543.s001.pdf]

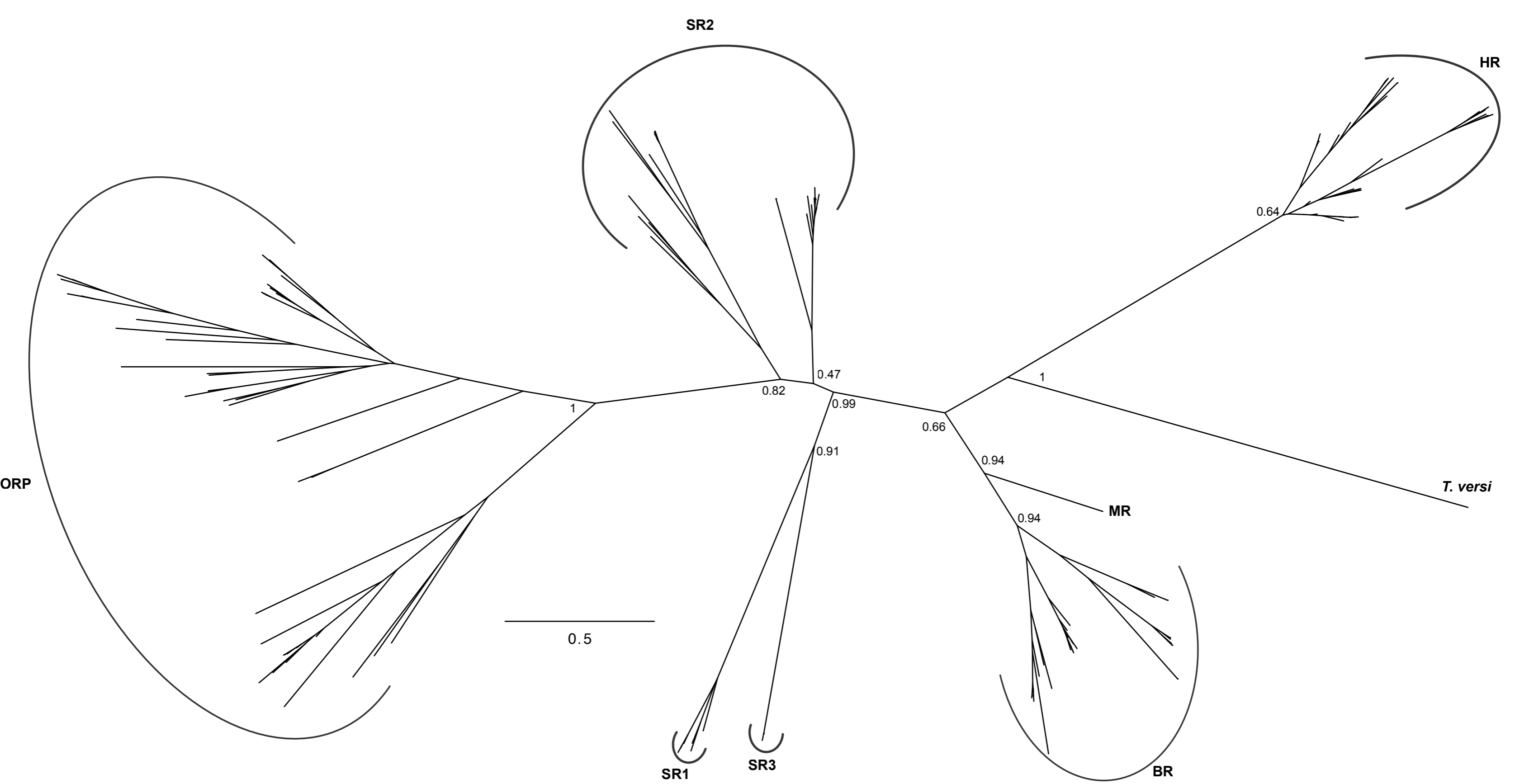

Supplement: S4 Fig — Unrooted maximum-likelihood phylogeny of sequences used for Fig 2C plus the single recovered haloarchaeal-type ORP from outside of the Haloarchaea. Tree inferred using FastTree [71]. (PDF) [file pone.0156543.s004.pdf]

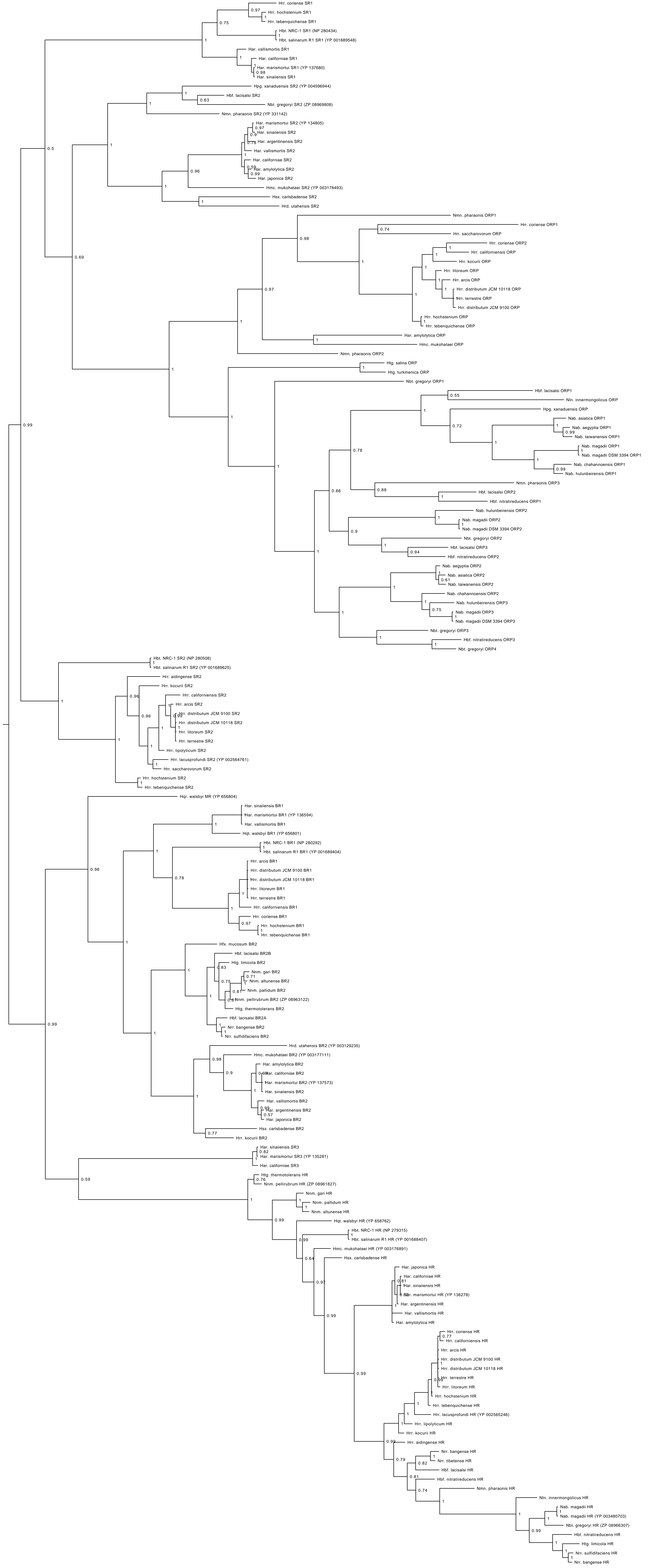

Supplement: S5 Fig — Phylogenetic tree of 170 haloarchaeal opsin proteins constructed using Bayesian inference with MrBayes [42]. Abbreviations as in Fig 1. Tree file can be accessed at the Dryad Digital Repository (http://dx.doi.org/10.5061/dryad.963hr). (PDF) [file pone.0156543.s005.pdf]

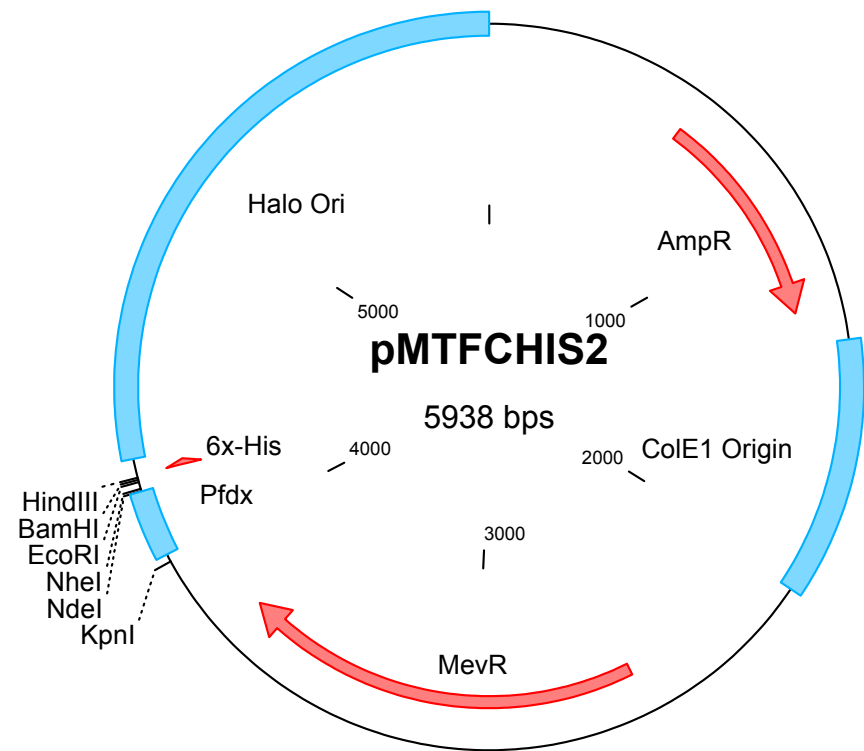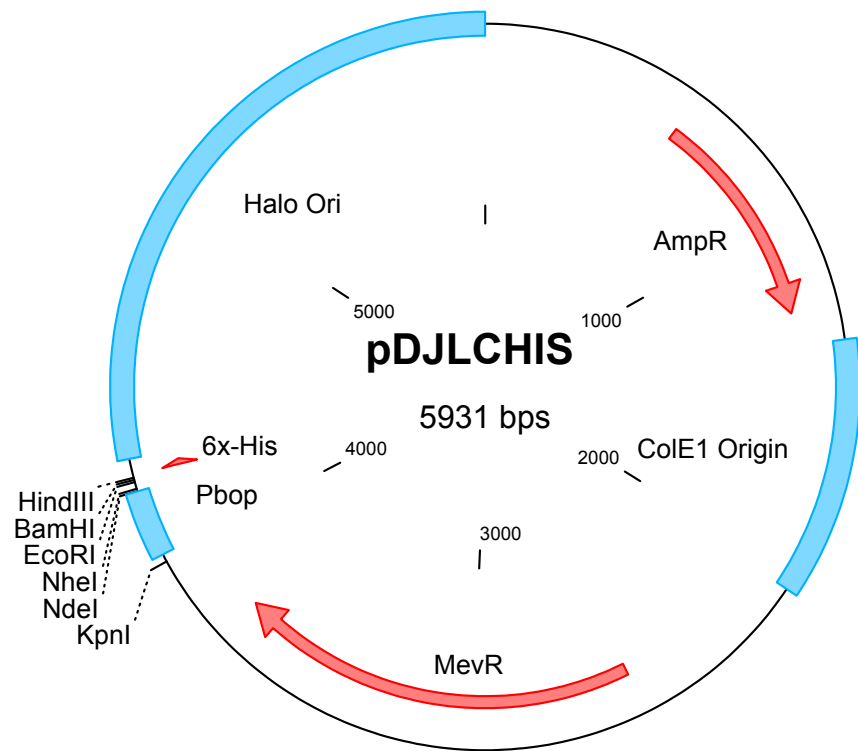

Supplement: S6 Fig — Plasmid maps of heterologous expression vectors pDJLCHIS and pMTFCHIS2. Vectors differ only in promoter driving expression of inserted gene. Pfdx = ferredoxin promoter, Pbop = bop promoter. Plasmid sequences available at the Dryad Digital Repository (http://dx.doi.org/10.5061/dryad.963hr). (PDF) [file pone.0156543.s006.pdf]

# B

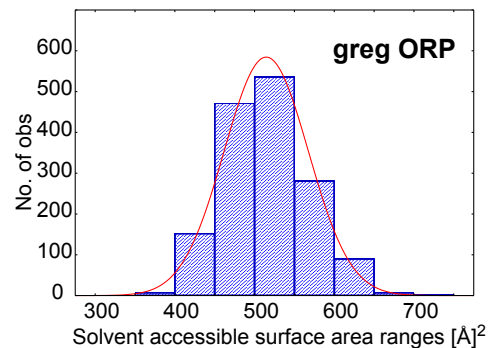

# B

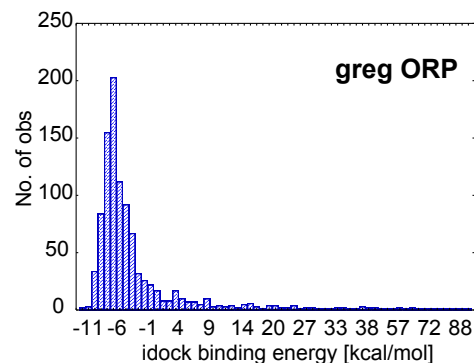

C

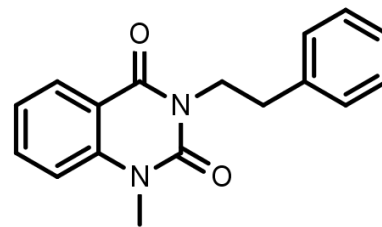

**quinazolinedione**  
**(-9.15 kcal/mol)**

Supplement: S7 Fig — A Histograms for solvent accessible surface area ranges [Å]2 for compounds with binding energies less than -9 kcal/mol. B idock binding energies for 229,358 ligands from natural product space. Compounds with binding energies less than -9 kcal/mol were selected for further analysis. C Representative compounds identified during virtual compound screening. These compounds include naphthoquinones, nitrogen-containing heterocycles and a number of sesquiterpenes. The name or compound class is given, as well as the docking energy for each single compound with one of the three modeled ORPs. (PDF) [file pone.0156543.s007.pdf]
