# Supplementary material for: A Large and Phylogenetically Diverse Class of Type 1 Opsins Lacking a Canonical Retinal Binding Site
Source: PLoS One. 2016 Jun 21;11(6):e0156543. doi: 10.1371/journal.pone.0156543 (PMC4915679; doi:10.1371/journal.pone.0156543)
Supplement: S1 File — Additional discussion regarding the results of the virtual ligand screening performed herein are described. (PDF) [file pone.0156543.s008.pdf]

Supplementary Discussion: Virtual Ligand Screening

AutoDock VINA and the idock software use similar scoring functions that are based on intermolecular and intramolecular contributions including steric interactions, repulsion, hydrophobic and hydrogen binding terms [1, 2]. The user-submitted partial charges as well as the nonpolar hydrogens are ignored. For a given ligand, different conformations are tested and the final result is given as predicted free energy of binding (kcal/mol). VINA and idock are multithreaded, however idock allows for an up to 3-fold speedup in terms of CPU time [1] as well as processing of multiple ligands in a time-efficient manner.

The idock threading efficiency was tested with different thread counts ranging from 16-64 threads. The performance for the dual socket 8-core, 32 threads machine peaked at 32 threads with 5.79 ligands per second. Under-using threads the efficiency fell to 5.13 ligands per second and assigning twice the physical available threads the efficiency fell to 4.03 ligands per second. The disk read/write overhead using a RamDisk was extremely low, hence boosting overall performance. In comparison, when the benchmark was performed on a normal disk system it was 45% slower. However this estimation is highly dependent on the docking parameters such as pocket size and conformer complexity. Overall, the ligand database of 229,358 compounds could be screened in around 11 hours.

The Universal Natural Products Database [3] comprises one of the largest and most diverse [4] publicly available datasets of natural compounds from plants, fungi and microorganisms. The compound classes in UNPD and other large databases such as the Dictionary of Natural Products (http://dnp.chemnetbase.com) cover several compound classes including aliphatic compounds, polyketides, carbohydrates, oxygen heterocycles, simple aromatic natural products,

benzofuranoids, benzopyranoids, flavonoids, tannins, lignans, polycyclic aromatics, terpenoids, steroids, amino acids and peptides, alkaloids and polypyrroles. We deliberately chose to confine our search to natural products because larger screening datasets commonly also include organic synthetic chemicals, but such xenobiotic compounds may be less useful for biological interpretations. In order to find energy-cutoff values for further statistical evaluations, we performed an independent virtual screening run with known binding ligands and decoy compounds. The energy cut-off was set at a binding energy of -9 kcal/mol (lower energy indicates better binding).

The binding energy distributions are given in S7 Fig panel B. An overlap analysis performed with the docking IDs resulted in 92 candidates for *Nab. magadii* WP_004267173, 495 candidates for *Hrr. distributum* ELZ45759 and 1547 candidates for the *Nbt. gregoryi* WP_005575895. An overlap analysis of the compound IDs showed that no individual docked ligand was shared among all three modeled ORPs. However a compound class based analysis showed many compound scaffolds were indeed overlapping.

S7 Fig panel C shows high ranking ligands from each of the three homology opsins. The scaffold for these naphthoquinone derivatives includes oxygen containing heterocycles and some of these compounds are naturally occurring antibiotics. Several other similar compounds were included as high ranking members of the screening list. The quinazolinedione is a representative of nitrogen-containing heterocycles and such natural products are widely occurring in nature [5]. S7 Fig also shows mikamicranolide which is a member of the germacrane sesquiterpene class. These are widely available bioactive compounds found in insects and plants [6]. The toxicity and bioactivity of some of these compounds may represent barriers for experimental applications.

Nevertheless the screening approach itself is compound-class agonistic and is driven by the physicochemical parameter space of the compounds themselves. A statistical analysis of some of the compound descriptors showed the commonly observed high correlation between molecular weight, molecular surface area and molecular volume. Each of the top scoring molecules fell into a specific range for each of these compound descriptors. A non-linear relationship was observed between those descriptors and the docking energies. However, the large diversity did not allow for a simple parametric equation to quickly establish high-affinity binding. The solvent accessible surface area ranges $[Å]^2$ for high-affinity binding ligands (lower than -9 kcal/mol) are shown in SI Fig 7a. For *Nab. magadii* WP_004267173 a total of 92 cases were retained with 466 $(Å)^2$/386 $(Å)^2$/581 $(Å)^2$ (mean/min/max). For *Hrr. distributum* ELZ45759 overall 492 candidates remained with 522 $(Å)^2$ /357$(Å)^2$/547$(Å)^2$ (mean/min/max). And finally for *Nbt. gregoryi* WP_005575895 a total number of 1547 high affinity molecules were retained with 514 $(Å)^2$/351 $(Å)^2$/716 $(Å)^2$ (mean/min/max). All of the observed scaffolds or compound core ring systems fell into these ranges.

Virtual screening results from docking programs should be interpreted as preliminary hypotheses. Benchmarks have shown that “virtually no correlations could be observed between the docking score and *in vitro* binding affinities” [7]. However it is important to mention that the predicted rankings of screened compounds can be in good agreement with the rankings from experimental binding affinity predictions [8]. It is also important to keep in mind that structural models are based on one particular state of the template structure and that other conformational states of the ORPs may lead to alternate ligand predictions which may or may not overlap between the three modeled ORPs.

With the availability of large screening sets and highly accurate crystal structures [9], deeper insights into the mode of action of transmembrane transporters and their ability to distinguish between different ligands such as monoamines, nucleosides, lipids or peptides are now possible [10]. Here we laid out the theoretical framework for future experiments, because the ultimate litmus test for virtual screening approaches is always an experimental validation of successful docking experiments [11].

Supplemental References

1. Li H, Leung K-S, Wong M-H. idock: A multithreaded virtual screening tool for flexible ligand docking. Comput Intell Bioinforma Comput Biol (CIBCB), 2012 IEEE Symp. 2012; 77–84.
2. Trott O, Olson A. AutoDock Vilna: improving the speed and accuracy of docking with a new scoring function, efficient optimization, and multithreading. J Comput Chem. 2010;31: 455–461.
3. Gu J, Gui Y, Chen L, Yuan G, Lu HZ, Xu X. Use of Natural Products as Chemical Library for Drug Discovery and Network Pharmacology. PLoS One. 2013;8: 1–10. doi:10.1371/journal.pone.0062839
4. Berkov S, Mutafova B, Christen P. Molecular biodiversity and recent analytical developments: A marriage of convenience. *Biotechnol. Adv.* 2014. **32:** 1102–1110.
5. D'yakonov A & Telezhenetskaya M. Quinazoline alkaloids in nature. Chem. Nat. Compd. 1997. **33:** 221–267.
6. Tashkhodzhaev B and Abduazimov B. Stereochemistry of sesquiterpenes of the germacrane

type. Chem. Nat. Compd.1997. **33:** 382–388.

7. Plewczynski D, Lazniewski M, Augustyniak R & Ginalski K. Can we trust docking results? Evaluation of seven commonly used programs on PDBbind database. J. Comput. Chem. 2011. **32:** 742–755.
8. Suenaga A, Okimoto N, Hirano Y, Fukui K. An efficient computational method for calculating ligand binding affinities. PLoS One 2012. **7:** (8):e42846
9. Choe HW, Park JH, Kim YJ, Ernst OP. Transmembrane signaling by GPCRs: Insight from rhodopsin and opsin structures. Neuropharmacology 2011. **60:** 52–57.
10. Granier S & Kobilka B. A new era of GPCR structural and chemical biology. Nat. Chem. Biol. 2012. **8:** 670–673.
11. Waszkowycz B, Clark D, Gancia E.Outstanding challenges in protein–ligand docking and structure−based virtual screening. Wiley Interdiscip. Rev. Comput. Moelcular Sci. 2011. **1:** 229–259.
