# Supplementary material for: A Large and Phylogenetically Diverse Class of Type 1 Opsins Lacking a Canonical Retinal Binding Site
Source: PLoS One. 2016 Jun 21;11(6):e0156543. doi: 10.1371/journal.pone.0156543 (PMC4915679; doi:10.1371/journal.pone.0156543)
Supplement: S1 Table — Strain names and number of ORP homologs detected for each haloarchaeal species possessing putative retinal-free opsins. (PDF) [file pone.0156543.s009.pdf]

SI Table 1: Haloarchaeal species with ORPs.

Species	# ORP homologs
Haloarcula amylolytica JCM 13557	1
Halobiforma lacisalsi AJ5	3
Halobiforma nitratireducens JCM 10879	3
Halomicrobium mukohataei DSM 12286	1
Halopiger xanaduensis SH-6	1
Halorubrum arcis JCM 13916	1
Halorubrum californiensis DSM 19288	1
Halorubrum coriense DSM 10284	2
Halorubrum distributum JCM 9100	1
Halorubrum distributum JCM 10118	1
Halorubrum hochstenium ATCC 700873	1
Halorubrum kocurii JCM 14978	1
Halorubrum litoreum JCM 13561	1
Halorubrum saccharovorum DSM 1137	1
Halorubrum tebenquichense DSM 14210	1
Halorubrum terrestre JCM 10247	1
Haloterrigena salina JCM 13891	1
Haloterrigena turkmenica DSM 5511	1
Natrialba aegyptia DSM 13077	2
Natrialba asiatica DSM 12278	2
Natrialba chahannoensis JCM 10990	2
Natrialba hulunbeirensis JCM 10989	3
Natrialba magadii ATCC 43099	3
Natrialba magadii DSM 3394	3
Natrialba taiwanensis DSM 12281	2
Natronobacterium gregoryi SP2	4
Natronolimnobius innermongolicus JCM 12255	1
Natronomonas pharaonis DSM 2160	3
