# Supplementary material for: A Large and Phylogenetically Diverse Class of Type 1 Opsins Lacking a Canonical Retinal Binding Site
Source: PLoS One. 2016 Jun 21;11(6):e0156543. doi: 10.1371/journal.pone.0156543 (PMC4915679; doi:10.1371/journal.pone.0156543)
Supplement: S2 Table — Search statistics and results for search for haloarchaeal-type ORPs. For details on filtering parameters, see Methods and Materials. Briefly, databases were searched using all haloarchaeal ORPs as query, and results were filtered for unique hits containing a Bac_rhodopsin domain (Pfam clan CL0192). Results matching these criteria were aligned and checked for presence or absence of the Schiff base lysine (K216). (PDF) [file pone.0156543.s010.pdf]

SI Table 2: Databases searched for haloarchaeal-type ORPs.

Database	Date	# seqs in database	BLASTp parameters	# unique hits to ORPs	# hits with rhodopsin domain	# hits with rhodopsin domain lacking K-216
CAMERA: MarineMicrobes	12/18/12	714,298	e = 1, max seqs = 50000			
CAMERA: All Metagenomic ORF Peptides from Sanger reads	12/18/12	43,240,119	e = 1, max seqs = 50000			
CAMERA: HOT	12/18/12	449,086	e = 1, max seqs = 50000			
CAMERA: GOS	12/18/12	41,146,566	e = 1, max seqs = 50000			
CAMERA: Combined Assembly Proteins	12/18/12	6,115,750	e = 1, max seqs = 50000			
CAMERA: move858 Assembly Proteins	12/18/12	11,860	e = 1, max seqs = 50000			
**CAMERA: TOTAL**		**91,677,679**		**127**	**5**	**0**
**NCBI: env_nr**	**12/18/12**		**e = 1, max seqs = 100000**	**8**	**4**	**0**
**NCBI: nr**	**12/18/12**		**e = 1, max seqs = 100000**	**210**	**199**	**1 (fungal)**
