# Supplementary material for: A Large and Phylogenetically Diverse Class of Type 1 Opsins Lacking a Canonical Retinal Binding Site
Source: PLoS One. 2016 Jun 21;11(6):e0156543. doi: 10.1371/journal.pone.0156543 (PMC4915679; doi:10.1371/journal.pone.0156543)
Supplement: S3 Table — PCR and cloning primers for ORPs from four species used in experimental confirmation of ORP expression. Expected product lengths and primer set used to screen for gDNA contamination also provided. (PDF) [file pone.0156543.s011.pdf]

SI Table 3: Primer sequences.

	Target gene	Forward primer name	Forward primer 5'--3'	Reverse primer name
1	*Hrr. distributum* 00779 (RFO)	Fdis61	GGTACTGCTACCCGTTCGTC	Rdis61
2	*Hrr. distributum* 00779 (RFO)	Fdis63	GGCAACTGCTCAGCGACTA	Rdis63
3	*Hrr. litoreum* 01500 (RFO)	Flit31	GGTACTGCTACCCGTTCGTC	Rlit31
4	*Hrr. litoreum* 01500 (RFO)	Flit34	GCGGATCGGGTACGACTTC	Rlit34
5	*Nab. magadii* 00584 (RFO1)	Fmag91	CGAATCAGTACGGCTACGCT	Rmag91
6	*Nab. magadii 0*0584 (RFO1)	Fmag94	GTGGGACAACTGGTCGTGAT	Rmag94
7	*Nab. magadii* 01352 (RFO3)	Fmag101	GGTCTTCCTGCTGTGGACTC	Rmag101
8	*Nab. magadii* 01352 (RFO3)	Fmag104	TGATGACAGCGGTCTTCCTG	Rmag104
9	*Nab. magadii* 01354 (RFO2)	Fmag113	AGTCCTCATCCTGGTGTCGT	Rmag113
10	*Nbt. gregoryi* 00694 (RFO4)	Fgreg153	CGAACACGGCACACTCTTTG	Rgreg153
11	*Nbt. gregoryi* 00695 (RFO3)	Fgreg166	GCTTCCCTCGTTCTTCTCGT	Rgreg166
12	*Nbt. gregoryi* 01746 (RFO2)	Fgreg182	TTACTCGGGCTAGTAGCGGT	Rgreg182
13	*Nbt. gregoryi* 03090 (RFO1)	Fgreg191	GGCAAACGAGATCCTGACCA	Rgreg191
14	*Hrr. distributum* 00779 (RFO)	c_Hrr_dist_lit_00779 /01500_F	ACCAAGCTTGTCCGCGTTCGACG	c_Hrr_dist_00779_R_fix
15	*Nab. magadii* 01354 (RFO2)	c_Nat_mag_01354_F	ATTCATATGATCGCGAGCGAAACG	c_Nat_mag_01354_R_fix
16	*Har. marismortui* YP_137573	c_KS_Hmar137573.1_F	ATTCATATGCCAGCACCAGGGA	c_KS_Hmar137573.1_R
17	*Hbt. sp.* NRC-1 *pstC2* VNG0455G	c_PstC2_NdeI_F	GACAAGCATATGATGAGCGGAGACGACC	c_PstC2_6His_XhoI_R
18	pET29b+ Clones	s_T7_Promoter_Primer	TAATACGACTCACTATAGGG	s_T7_Terminator_Primer
19	Haloarchaeal 16S rDNA	Halo_F_F02	GGCCTAAAGCGTCC	Archaeal_and_Halo_R_1492Ra

	Reverse primer 5'--3'	Product length (bp)	Use
1	GTACTCTAAGGTCACCGCCG	488	Native expression
2	TGAACGTATCGAACAGCCCG	357	Native expression
3	AGGAGGTCGAGGTACTCCAG	499	Native expression
4	CGAGGTACTCCAGCGTCAC	272	Native expression
5	ATCACGACCAGTTGTCCCAC	484	Native expression
6	GCTCGCTCTACTGAGTCACC	260	Native expression
7	TACGGTCGCCCAGAGACTTA	280	Native expression
8	TCAGATTCTTGAGTCGCCCG	450	Native expression
9	GTTCCGAAAGAGGAGCAAGC	265	Native expression
10	TCAGGAACTTGAGCTTGGCA	494	Native expression
11	ACCTCTTCTGCCTCGAGTGT	296	Native expression
12	TCTGCGGTCCCAGAAAGAAC	329	Native expression
13	GGTCACCGAGAGATTGCGAA	346	Native expression
14	ATTCATATGGTGCGGTTCGCCTCCG	723	Cloning
15	ACCAAGCTTCCTGCCGCTCACAC	744	Cloning
16	ACTAAGCTTGTCGTCTGCAGGCGT	753	Cloning
17	AGAATGCTCGAGTCAGTGGTGATGA TGGTGATGGTACTCCTCCTTGAA	960	Cloning
18	GCTAGTTATTGCTCAGCGG	Variable	pET29b+ specific
19	ACGGHTACCTTGTTACGACTT	Variable	gDNA contamination
