# Supplementary material for: A Large and Phylogenetically Diverse Class of Type 1 Opsins Lacking a Canonical Retinal Binding Site
Source: PLoS One. 2016 Jun 21;11(6):e0156543. doi: 10.1371/journal.pone.0156543 (PMC4915679; doi:10.1371/journal.pone.0156543)
Supplement: S2 Fig — Output visualizations from CAVER [58] showing predicted novel extracellularly accessible binding pocket and internal tunnel network for top five structural models predicted for Hrr. distributum ELZ45759, Nab. magadii WP_004267173, Nbt. gregoryi WP_005575895. (PDF) [file pone.0156543.s002.pdf]

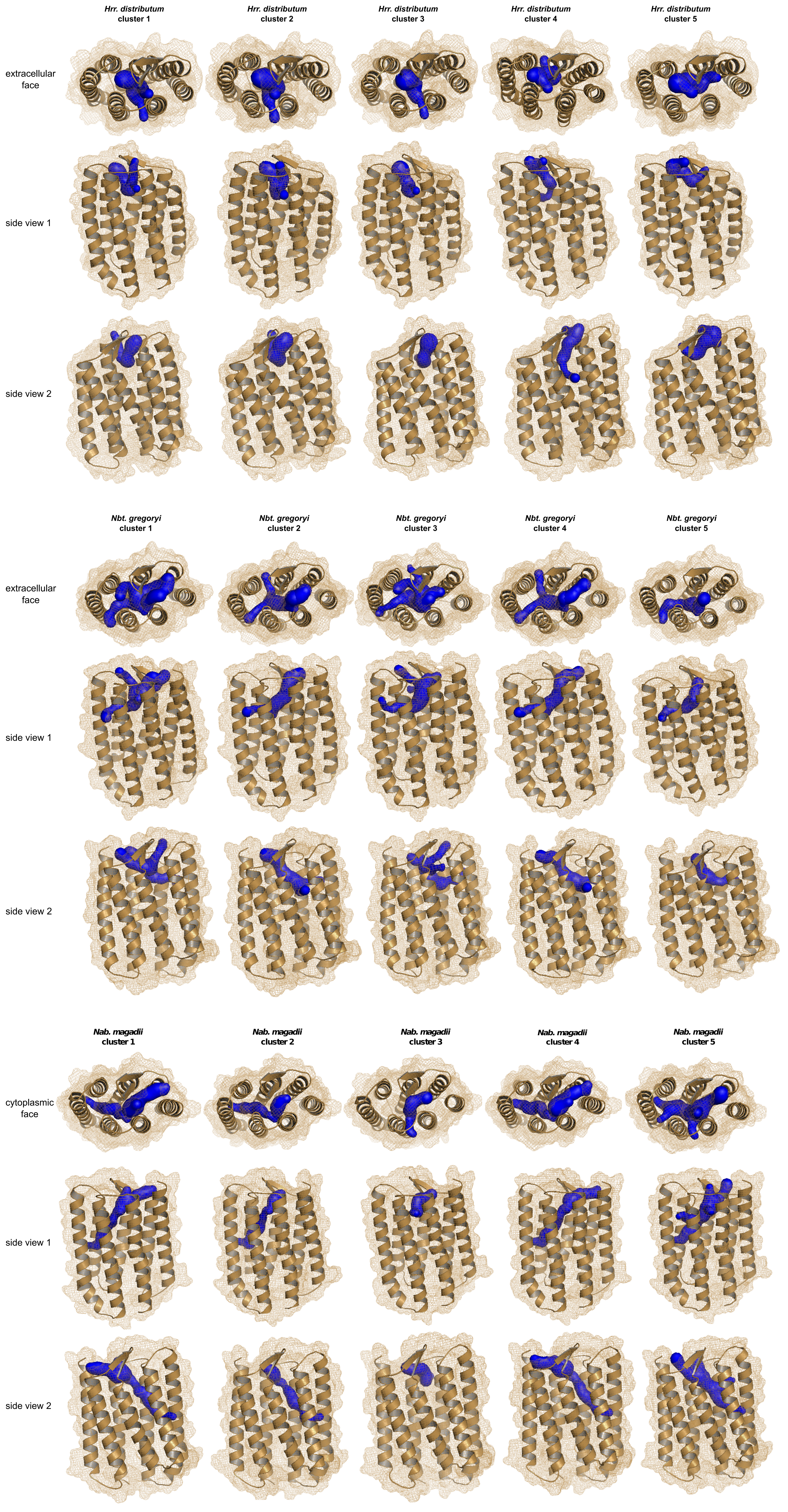

Hrr. distributum cluster 1
Hrr. distributum cluster 2
Hrr. distributum cluster 3
Hrr. distributum cluster 4
Hrr. distributum cluster 5
extracellular face
side view 1
side view 2
Nbt. gregoryi cluster 1
Nbt. gregoryi cluster 2
Nbt. gregoryi cluster 3
Nbt. gregoryi cluster 4
Nbt. gregoryi cluster 5
extracellular face
side view 1
side view 2
Nab. magadii cluster 1
Nab. magadii cluster 2
Nab. magadii cluster 3
Nab. magadii cluster 4
Nab. magadii cluster 5
cytoplasmic face
side view 1
side view 2
