# Supplementary material for: A Large and Phylogenetically Diverse Class of Type 1 Opsins Lacking a Canonical Retinal Binding Site
Source: PLoS One. 2016 Jun 21;11(6):e0156543. doi: 10.1371/journal.pone.0156543 (PMC4915679; doi:10.1371/journal.pone.0156543)

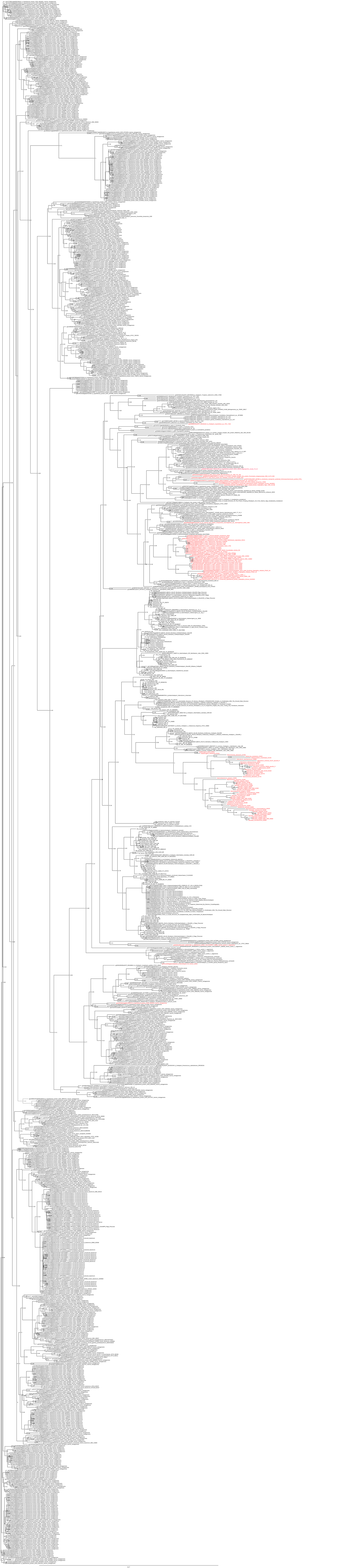

Supplement: S3 Fig — Unrooted maximum-likelihood phylogeny of all microbial (type 1) opsins obtained by BLASTp searches of NCBI’s nr and env_nr databases using canonical haloarchaeal opsins as queries. Tree inferred using FastTree [71] and ComparetoBootstrap.pl [74] using 500 bootstrap replicates generated with SeqBoot [73]. Sequences lacking the Schiff base lysine (K216) are colored red. Tree file can be accessed at the Dryad Digital Repository (http://dx.doi.org/10.5061/dryad.963hr). (PDF) [file pone.0156543.s003.pdf]
